# Rare Cause of Lower Gastrointestinal Bleeding—Case Presentation and Literature Review

**DOI:** 10.3390/reports8020082

**Published:** 2025-06-01

**Authors:** Cristian Iorga, Cristina Raluca Iorga

**Affiliations:** 1 Faculty of Medicine, “Dr. Carol Davila” University of Medicine and Pharmacy, 050474 Bucharest, Romania; cristina.iorga@umfcd.ro; 2 Surgery Clinic, “Dr. Carol Davila” Clinical Nephrology Hospital, 010731 Bucharest, Romania

**Keywords:** rare cause, lower gastrointestinal bleeding, case report

## Abstract

**Background and Clinical Significance:** Gastrointestinal bleeding is a critical medical emergency, with upper gastrointestinal bleeding occurring approximately five times more frequently than lower gastrointestinal bleeding (LGIB). The incidence of LGIB tends to increase with age, likely due to a greater prevalence of vascular and diverticular diseases among older patients. However, there are rare or extremely rare causes of LGIB that demand significant diagnostic and therapeutic efforts, some of which may pose unexpected challenges during surgery. Case report: We present the case of a 75-year-old woman, previously treated for a cecal neoplasm 15 years ago, who was hospitalized due to intermittent lower gastrointestinal bleeding over the past three months. Initially, the patient declined a colonoscopic examination, and the bleeding stopped spontaneously. She was then discharged at her own request in stable condition. However, she returned with a recurrence of the bleeding. While preparing for a colonoscopy, she experienced subocclusive symptoms, abdominal distension, and vomiting. During emergency surgery, a floating coprolith, which was attached to one of the anastomosis sutures, was sensed through palpation and later confirmed via colotomy. The coprolith was removed, and hemostasis was achieved in situ, leading to a favorable postoperative recovery and normalization of intestinal transit. A literature review identified 24 articles that met the eligibility criteria concerning rare causes of LGIB. Appendiceal bleeding (due to erosions, arteriovenous malformations, or endometriosis) was the most common cause, whereas the rarest causes included jejunal hemangiomas and rectal ulcers resulting from mucormycosis. Diagnosing these conditions is often challenging, typically requiring CT scans, colonoscopy, and angiography, with surgical treatment being the primary method to ensure hemostasis. In conclusion, the diagnosis and management of LGIB present significant challenges for clinicians, and successful outcomes are usually achieved through a collaborative multidisciplinary team approach.

## 1. Introduction and Clinical Significance

Lower gastrointestinal bleeding (LGIB) is a significant pathology in emergency admissions, accounting for approximately 30–40% of all gastrointestinal bleeding cases in the USA [1,2]. The most common causes of LGIB, in order of prevalence, include diverticular disease, angiodysplasia, ischemic colitis, colorectal neoplasms, hemorrhoidal disease, bleeding after polypectomy, post-radiotherapy enteritis, and gastrointestinal inflammatory disease [3,4]. Less common causes encompass colorectal varices, intussusception, Meckel’s diverticulum, and aortoenteric fistula [3,4].

The mortality rate in people with LGIB is 2.4–3.9% and can reach 40% in elderly patients with multiple associated comorbidities [5].

This has led to ongoing concerns about accurately diagnosing the cause of GI bleeding, particularly when selecting the appropriate treatment. In the early 1900s, the diagnosis and treatment of LGIB were almost exclusively the responsibility of surgeons. However, gastroenterologists have become increasingly involved with the introduction of diagnostic and interventional endoscopic procedures. Today, in addition to endoscopic techniques, new treatment methods have emerged, including those from interventional radiology and nuclear medicine, while surgery remains a last resort when other methods are no longer effective [5,6,7,8,9]. The diverse array of diagnostic and treatment options has led to a tailored approach for managing LGIB patients, especially concerning treatment and diagnosis [10]. Despite the significant advancements that have been made in recent years, the diagnosis and treatment of LGIB continue to spark various controversies, primarily due to the unusual or extremely rare causes of these hemorrhages. Such cases necessitate close collaboration among multidisciplinary medical teams, including ICU staff, gastroenterologists, radiologists, and surgeons, to accurately establish the diagnosis and the most appropriate treatment strategy.

## 2. Case Presentation

A 75-year-old female patient was referred to the General Surgery Clinic of Dr. Carol Davila Nephrology Hospital in Bucharest due to the appearance of bloody stools two days prior to admission.

The patient was hemodynamically stable but presented with anemia (hemoglobin 10.2 g/dL), hypoalbuminemia, and hypoproteinemia. All other laboratory test values were normal.

Upon admission, the patient was in poor general condition, had a pale complexion, and exhibited a soft, painless abdomen with stools that appeared reddish-burgundy. She has a history of cecal neoplasm, which was surgically treated 15 years ago (right hemicolectomy with manual anastomosis), followed by six chemotherapy sessions, and was declared cured after five years of oncological follow-up (documents unavailable). There are no chronic illnesses, and she is not receiving any chronic treatment. Over the past three months, she has experienced abdominal discomfort characterized by distention and intermittent colicky pain, accompanied by diarrhea. She has had 2–3 episodes of rectorrhea, which were self-limiting. The current episode started 2 days ago, with repeated stools (3–4 times a day) exhibiting a bloody, vein-like appearance. She was admitted for diagnostic and therapeutic management.

Because the patient’s history includes a right hemicolectomy with ileo-transverse manual termino-lateral anastomosis for cecal neoplasm, performed 15 years ago, tumor markers CA 19-9 and carcinoembryonic antigen—both of which showed normal results—were also collected. According to the patient’s reports, not every stool was bloody, and the last stool was no longer red. After admission, the next stool appeared normal, and the patient was advised to start a digestive preparation for colonoscopy, which she refused. During the hospitalization (5 days inpatient), the patient’s stool remained normal, leading to the conclusion. 

According to the patient’s reports, not every stool was bloody, and the last stool was no longer red. After admission, the next stool appeared normal, and the patient was advised to start a digestive preparation for the colonoscopy, which she refused. During the hospitalization of five days, the stool remained normal, leading to the conclusion that the bleeding stopped spontaneously, as is common in many cases of LGIB.

After approximately three months, the patient returned to our clinic, again presenting with bloody stools and hematochezia. This time, the hemoglobin level was lower (8.7 g/dL), and the patient was admitted to the ICU with hypotension (blood pressure 80 mmHg). Upon clinical examination, there was moderate abdominal distension but no pain. A rectal exam revealed traces of red blood on the glove. Hydroelectrolyte rebalancing treatment was initiated, to which the patient responded positively. The patient was again recommended to undergo colonoscopy after appropriate bowel preparation; this time, she accepted.

During the digestive preparation, the patient experienced nausea and vomiting, which interrupted the ingestion of the purgative solution. As in the previous admission, the stool color normalized the following day. To establish a diagnosis, a computerized tomography (CT) examination with angiographic timing was recommended to identify the bleeding site.

**Figure 1:**
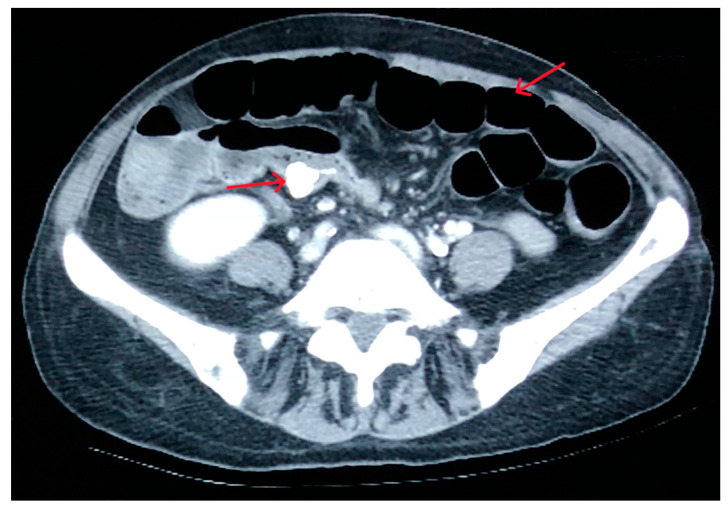
CT image with the foreign body (left arrow) and hydroaeric images (right arrow).

During the CT examination, it was not possible to determine the source of the hemorrhage, since the digestive bleeding had ceased. However, a 1.5 cm foreign body was observed in the colon (right iliac fossa) along with multiple hydroaeric images (Figure 1).

Later, the lower gastrointestinal hemorrhage resumed, resulting in stools with burgundy-colored blood and a 1 g/dL decrease in hemoglobin levels. In light of this situation, the decision was made to carry out an emergency surgical intervention to clarify the diagnosis and perform surgical hemostasis.

We performed surgery using an iterative median laparotomy and found a stenotic ileo-colic anastomosis accompanied by perianastomotic adhesions and dilated small bowel loops, with the colon appearing normal. Upon palpation, we detected a hard, approximately 2 cm floating mass within the colon. A colotomy was executed, focusing on the foreign body. Intraoperatively, we were surprised to identify the foreign body, as shown in the CT scan, located within the colonic lumen next to the ileocolic anastomosis, floating a few centimeters between the transverse colon and the terminal ileum.

At the same time, the ileocolic anastomosis was entangled in a perianastomotic adhesion process that partially twisted it, which explained the hydroaeric levels noted in the CT scan and the challenges in preparing the colon for the colonoscopic examination.

The foreign body was removed through colotomy and consisted of a coprolith attached to one of the sutures used for the anastomosis (silk thread) (Image 2). The mucosa of the colon and terminal ileum showed numerous erosions covered with blood clots, though there was no active bleeding during the surgery (biopsies taken). The histopathological findings are as follows: the pathological material includes multiple fragments of colonic mucosa with preserved morphology and mucous secretion, accompanied by significant lymphoplasmacytic inflammatory infiltration distributed both diffusely and in follicular lymphoid aggregates. No signs indicating malignancy were found.

**Figure 2:**
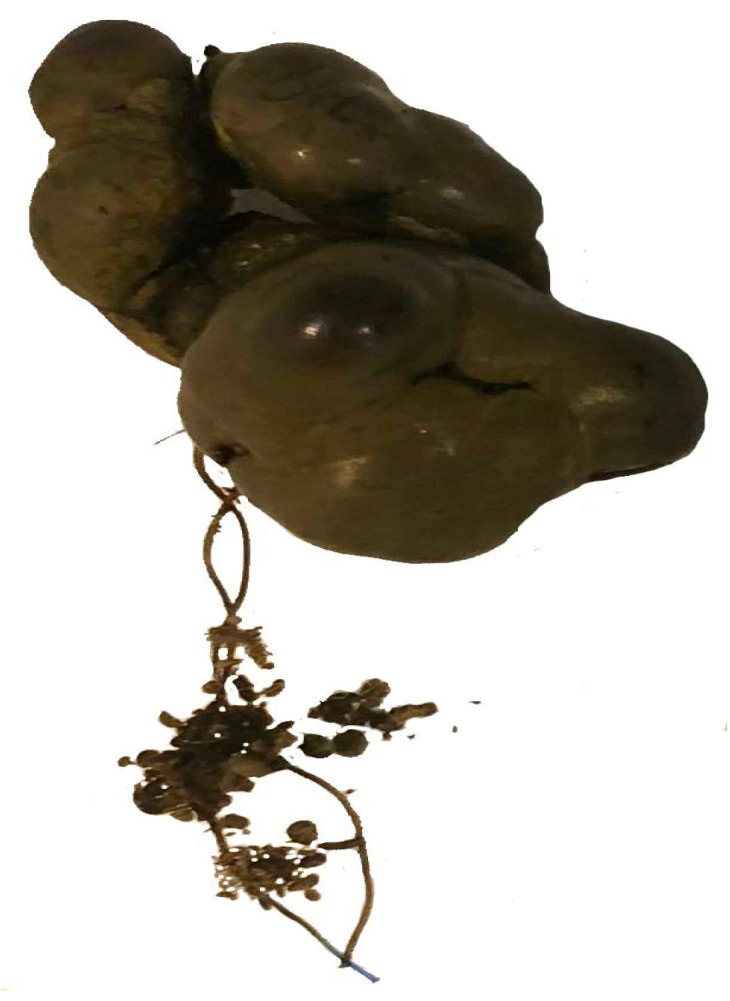
Coprolith attached to the suture thread.

Thus, the source of the digestive bleeding was identified, and definitive hemostasis was achieved by applying in situ hemostasis threads—polysorb. No 3/0 (Figure 2).

The patient’s postoperative recovery was positive, with digestive tolerance and bowel movement resuming by day 4. There were no signs of recurring lower gastrointestinal bleeding or obstruction. We followed up with the patient 5 years postoperatively and she was without signs of digestive disorder. Essentially, we viewed this case as a late complication of the postoperative period, with no indicators of neoplastic recurrence, resulting from the formation of a coprolith that eroded the intestinal mucosa and led to repeated bleeding.

## 3. Literature Review

We conducted a literature review by searching the following databases: PubMed, PubMed Central, Google Scholar, and Cochrane according to the following criteria: “rare” [All Fields] AND (“etiology” [Subheading] OR “etiology” [All Fields] OR “causes” [All Fields] OR “causality” [MeSH Terms] OR “causality” [All Fields]) AND lower [All Fields] AND (“gastrointestinal hemorrhage” [MeSH Terms] OR (“gastrointestinal” [All Fields] AND “hemorrhage” [All Fields]) OR “gastrointestinal hemorrhage” [All Fields] OR (“gastrointestinal” [All Fields] AND “bleeding” [All Fields]) OR “gastrointestinal bleeding” [All Fields]).

The search yielded 35,608 articles. The study took place from 15 October 2024 to 15 February 2025.

After the initial search, which produced a vast number of results (35,608), we attempted to refine it using the LGIB criteria and obtained 3247 results. The criteria remained the same, focusing on “lower gastrointestinal bleeding” while excluding “gastrointestinal hemorrhage”. It became apparent that some articles previously included in the review were no longer available, significantly decreasing the pool of eligible articles. Consequently, we decided to perform the narrative literature review following the first search.

The inclusion criteria were as follows: English full-text articles, case reports, and reports of rare causes of lower gastrointestinal bleeding. We excluded articles for which only abstracts were available, those not written in English, and studies that covered other digestive bleeding causes.

The criteria for inclusion and exclusion specified that articles must be in English, involve patients over 18 years of age, report on rare causes of LGIB, and include case presentations or series of cases.

## 4. Discussion

Only 24 articles meeting the eligibility criteria were identified [11,12,13,14,15,16,17,18,19,20,21,22,23,24,25,26,27,28,29,30,31,32,33,34].

Unexpectedly, the most frequently reported rare cases of LGIB involved appendiceal bleeding. We found 11 cases with various causes: mucosal erosion (4), arteriovenous malformation (3), and 1 case each of diverticula of the appendix, appendicular stump after appendectomy, appendicular endometriosis, and Dieulafoi’s lesion of the appendix. The diagnosis was established in most cases via colonoscopy; CT was rarely needed (2 cases). Even when appendiceal bleeding was confirmed colonoscopically, the final diagnosis was made through a pathological examination of the appendix following appendectomy. Laparoscopic appendectomy was the definitive treatment (unitary in all cases), although in two cases, endoscopic clipping was attempted [13,14,17,18,19,30,31].

Other rare causes of LGIB included anorectal hemangioma (3), colonic varices (2), colonic amyloidosis, Osler Rendu syndrome, jejunal hemangioma, Kasabach–Merrit syndrome, lung adenocarcinoma metastasis in the colon, inflammatory cloacogenic polyp mimicking rectal cancer, caecum lipoma, and rectal ulcer induced by mucormycosis. For diagnosing these rare causes of LGIB, the entire available arsenal was utilized: colonoscopy, CT, and angiography. In most cases, it was necessary to combine diagnostic methods to reach the final diagnosis—Colonoscopy + CT(5), Colonoscopy + Angiography(6), Colonoscopy + CT + Angiography (2). Thus, personalized treatment was tailored to the diagnosis of each case, with most requiring surgery (7), colonoscopic (3), and angiographic (1) hemostasis [15,16,20,21,22,23,24,25,26,27,28,29,34].

Most authors suggest utilizing the full range of diagnostic tools to establish the most accurate diagnosis, thereby ensuring the appropriate choice of treatment [14,17,20,21,22,23,24,27,31,32,34].

## 5. Conclusions

Rare causes of LGIB continue to present a significant challenge for clinicians, and establishing an accurate diagnosis requires a multidisciplinary team to ensure effective personalized treatment.

## Data Availability

The original contributions presented in this study are included in the article. Further inquiries can be directed to the corresponding author.

## Abbreviations

The following abbreviations are used in this manuscript:

CT	Computed tomography.
ICU	Intensive Care Unit.
GI	Gastrointestinal.
LGIB	Lower gastrointestinal bleeding.
